# A Method of FPGA-Based Extraction of High-Precision Time-Difference Information and Implementation of Its Hardware Circuit

**DOI:** 10.3390/s19235067

**Published:** 2019-11-20

**Authors:** Jian Li, Xinlei Yan, Maojin Li, Ming Meng, Xin Yan

**Affiliations:** 1 Shanxi Key Laboratory of Information Detection and Processing, North University of China, Taiyuan 030051, China; s1905115@st.nuc.edu.cn (X.Y.); s1905024@st.nuc.edu.cn (M.L.); s1708012@st.nuc.edu.cn (M.M.); s1605083@st.nuc.edu.cn (X.Y.); 2 National Key Laboratory of Electronic Measurement Technology, North University of China, Taiyuan 030051, China

**Keywords:** time difference, distributed source location, first break arrival time

## Abstract

The positioning technology to find shallow underground vibration sources based on a wireless sensor network is receiving great interest in the field of underground position measurements. The slow peaking and strong multi-waveform aliasing typical of the underground vibration signal result in a low extraction accuracy of the time difference and a poor source-positioning accuracy. At the same time, the transmission of large amounts of sensor data and the host computer’s slow data processing speed make locating a source a slow process. To address the above problems, this paper proposes a method for high-precision time-difference measurements in near-field blasting and a method for its hardware implementation. First, based on the broadband that is typical of blast waves, the peak frequency of the P-wave was obtained in the time–frequency domain, taking advantage of the difference in the propagation speed of the P-wave, S-wave, and the surface wave. Second, the phase difference between two sensor nodes was found by means of a spectral decomposition and a correlation measurement. Third, the phase ambiguity was eliminated using the time interval of the first break and the dynamic characteristics of the sensors. Finally, following a top-down design idea, the hardware system was designed using Field Programmable Gate Array(FPGA). Verification, using both numerical simulations and experiments, suggested that compared with generalized cross-correlation-based time-difference measurement methods, the proposed method produced a higher time-difference resolution and accuracy. Compared with the traditional host computer post-position positioning method, the proposed method was significantly quicker. It can be seen that the proposed method provides a new solution for solving high-precision and quick source-location problems, and affords a technical means for developing high-speed, real-time source-location instruments.

## 1. Introduction

**Figure 1:**
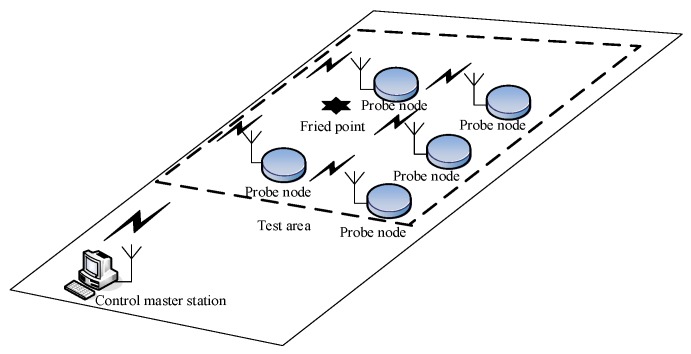
Schematic diagram of shallow ground distributed source localization.

Shallow subsurface distributed source localization involves a large number of sensor nodes that are buried at different depths in an underground near-field monitoring area, where a wireless sensor network is built up in a self-organized and multi-hop mode, which works in a collaborative way to sense, monitor, collect, process, and transmit the vibration signal generated from the source. Figure 1 shows the specific positioning process of the method. An upper computer then pre-processes the data, extracts the characteristics, builds up the location model, and solves the model to determine the location of the source. So far, this technology mainly relies on the deep seismic location method to construct, from the differences in time of the vibration signal reaching the nodes, i.e., using time difference of arrival (TDOA)-based source location equations [1,2,3], which are solved to determine the source location.

With the appearance of an increasing number of high-precision, quick shallow subsurface source location events (like the real-time positioning of high-value ammunition underground shot points; quick determination of fuse action positions; and advanced forecast of rockbursts, water inrushes, and mud inrushes), improving the real time performance of shallow subsurface source positioning is a critical technology calling for an urgent breakthrough.

So far, researchers have concentrated their efforts on how to improve the speed and accuracy of the TDOA positioning equations. For example, Salari et al. proposed using the Chan-Unscented Kalman Filter (UKF) algorithm as a way to reduce the influence of the initial value on the positioning accuracy [4,5]. Risoluti et al. proposed to reduce the local convergence in the TDOA solution process using an enhanced genetic algorithm (EGA) [6]. Gan et al. proposed using quantum-behaved particle swarm optimization (QPSO), which has a quicker global search, to improve the speed of positioning [7,8], and search traversal methods like the maximum likelihood and the spherical interpolation [9]. These algorithms are able to shorten the running time of the TDOA equations to tens of seconds, and the times do not vary much between these algorithms. However, in the positioning process, it is the transference of massive amounts of data, data pre-processing, and time-difference information extraction, which can be as long as tens of minutes, not the solving of the TDOA equations, that take up the larger part of the processing time. Lee et al. have studied data compression methods, such as compression sensing [10,11], yet for source localization, the upper computer only needs to know the time-difference information, a characteristic parameter for source location, in order to move on with source localization because in this process, the original vibration data are not necessary. Therefore, for the sake of quicker source positioning in real-time shallow source localization, it is critical to calculate the time difference on the sensor nodes and complete a quick wireless transference of the time-difference information.

Currently, generalized cross-correlation methods are generally used to extract time-difference information. They include generalized cross-correlation (GCC), Roth impulse response (RIR), smoothed correlation transform (SCOT) [12,13], phase transformation (PHAT), Hassab-Boucher (HB) weighting method and Hannan–Thomson (HT) [14,15,16]. These types of methods mainly measure the time of the two signal peaks as a way to determine the time difference and are suitable for extracting the information of the far-field time difference of the underground blasting. In shallow underground blasting, the distance to the explosion center is short, and the P-wave, S-wave, and surface wave are aliased. Because of this, the generalized cross-correlation methods give rise to grave misjudgment and take up a greater number of integrators and multipliers, making hardware implementation a hard task.

In view of the above problems, this paper explores high-precision time-difference measurement in near-field blasting and its hardware implementation. First, based on the broadband typical of blast waves and considering the difference of the propagation speeds of the P-wave, S-wave, and the surface wave, the peak frequency of the P-wave was obtained in the time–frequency domain. Second, the phase difference of the single cycle was found using spectral decomposition and correlation measurements. Third, the phase ambiguity was eliminated using the time difference of the first break and the dynamic characteristics of the sensors. Finally, following a top-down design idea, the hardware module was completed using FPGA. The proposed method provides a powerful theoretical solution for solving high-precision and quick source-location problems, and affords technical support for developing high-speed, real-time source-location instruments.

## 2. The Principle of High Precision Time-Difference Information Measurement

The shock wave generated by a subsurface blast produces a type of broadband, multispectral energy signal. Because of its physical properties, the soil medium behaves much like a bandpass filter, dampening higher frequency components as the vibration wave passes through this medium. However, some low-frequency components that fall within the soil’s passband can propagate for longer distances, and these frequency components are called the dominant frequencies of the vibration signal.

**Figure 2:**
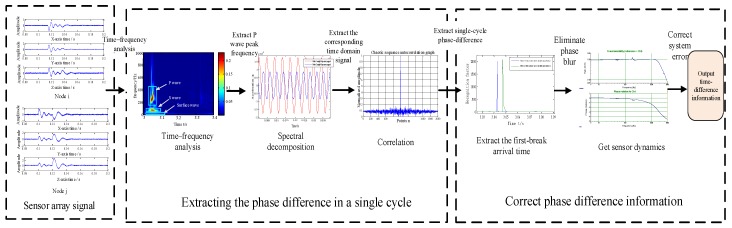
Flow chart of time-difference extraction.

According to Fourier’s theorem, a single-component time domain signal can be seen as a series of sinusoidal signals of different superimposed frequencies. For a single frequency signal, the corresponding time difference associated with this frequency component can be derived from the phase difference information between the two nodes under study. Although the time-difference information between the two nodes can be derived from the arrival time of the first break, the resolution of the phase difference is 360 times better than that of the corresponding signal period. Therefore, when the arrival time becomes an available piece of information, the time-difference resolution can be greatly improved through using the phase difference. The time-difference extraction process consists of three parts: extracting phase difference information between nodes, calibrating this information, and extracting time-difference information, as shown in Figure 2.

The extraction process of the time difference is shown in Figure 2. It involves extracting the phase difference of a single cycle between nodes, calibrating phase-difference information, and extracting the time difference. First, the peak frequency of the direct P-wave common to two sensor nodes is determined in the time–frequency domain through a Stockwell transformation (ST)-based time–frequency analysis [17], and the time-domain information corresponding to the frequency is extracted using a spectral decomposition. Second, the corresponding phase difference between the nodes is extracted using the cross-correlation algorithm. Third, the direct P-wave arrival time information of the two nodes is obtained through the short-term average/long-term average (*STA*/*LTA*) algorithm [18], and with the known geometry of the sensor array, the phase ambiguity is eliminated. Finally, the phase error is corrected by use of the sensor’s own dynamic characteristics to produce the required time-difference information.

### 2.1. Phase Difference Information Extraction Method Based on a Spectral Decomposition Technique

**Figure 3:**
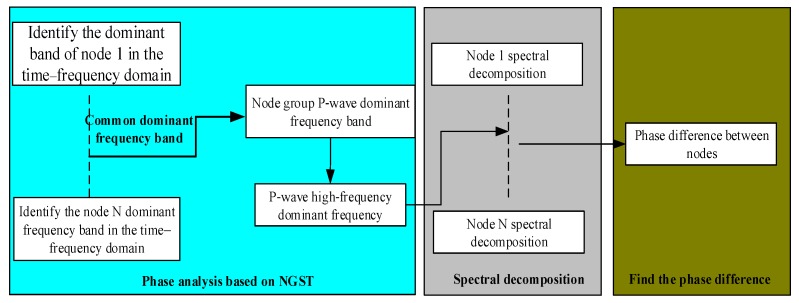
Flowchart of the phase-difference extraction.

Figure 3 shows the phase difference extraction procedure, which is made up of three steps. First, from the propagation speed difference between the P-wave, S-wave, and surface wave, the frequency range of the P-wave signal is determined in the time–frequency domain for each sensor. Second, the highest frequency of the P-wave common to all the sensors is taken as the frequency component for the phase-difference measurement of the system. Finally, spectral decomposition is performed in the frequency domain using the all-phase Fast Fourier Transform (FFT) to find the phase difference signal corresponding to the frequency component.

#### 2.1.1. Time–Frequency Domain Analysis to Determine the P-Wave’s Dominant Frequency Band Common to the Node Group

According to the wave theory [19,20], the P-wave travels at approximately $\sqrt{2}$ times the speed of the S-wave, and the speed of the surface wave is about 0.92 times that of the transverse wave. Therefore, after an underground blasting, the P-wave is the first to arrive at the sensor nodes.

First, from Equation (1), the acquired time domain signal is converted to the time–frequency domain through ST time–frequency analysis, where *h*(*t*) is the time series signal acquired by the sensor node:

(1) $$s(\tau ,f)=\frac{\left|f\right|}{\sqrt{2\pi }}\int_{-\infty }^{\infty }h(t)e^{-\frac{{\left(t-\tau \right)}^{2}f^{2}}{2}}e^{-j2\pi ft}dt.$$

**Figure 4:**
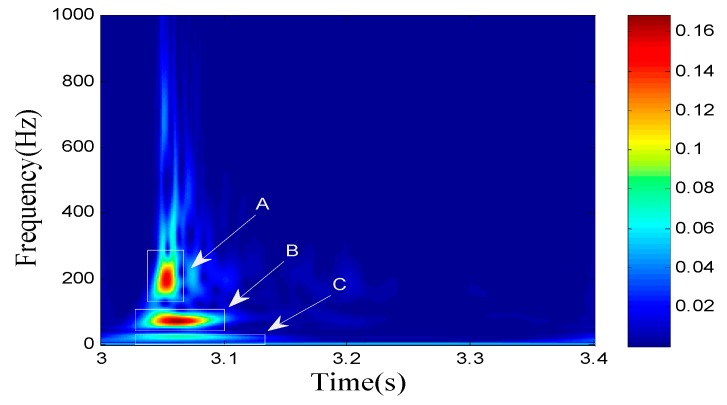
Time–frequency diagram of the node group (A is the P-wave region, B is the S-wave region, and C is the surface wave region).

Figure 4 shows a typical time–frequency diagram of the group wave in the near field of the blasting. From the time–frequency diagram [21,22], the P-wave frequency range corresponding to each node is determined in the time–frequency domain, which relies on the propagation speed dominance and frequency dominance of the P-wave.

Finally, in the above P-wave frequency range, the peak frequency common to all nodes is determined.

#### 2.1.2. Decompose Spectrally the Highest Dominant Frequency of P-Wave

Suppose that the highest dominant frequency of the P-wave is $f_{0}$. From the Fourier transform, its frequency domain expression is Equation (3):

(2) $$x(t)=\sin w_{0}t,$$

(3) $$F(\sin w_{0}t)=-j\pi \delta (w+w_{0})+j\pi \delta (w-w_{0}).$$

As can be seen from Equation (3), the spectrum of the discrete-time sinusoidal signal of the $w_{0}$ frequency is the two impulse pulses at point $w_{0}$ and $-w_{0}$. In order to extract the spectral signal and the time domain waveform of the highest dominant frequency $w_{0}$, Equation (4) is derived from Equation (3):

(4) $$F_{i}(w_{0})=\sum_{-\infty }^{\infty }\left(F_{i}(w).\delta (w-2\pi f_{0})+F_{i}(w).\delta (w-2\pi (F_{s}-f_{0}))\right)$$

According to Equation (4), the $\frac{f_{0}}{f_{s}}N_{\mathrm{length}}+1$ spectral line information corresponding to $w_{0}$ is extracted in the wide spectrum (where $N_{\mathrm{length}}$ is the total length of the vibration signal acquired by the sensor node, where $\frac{f_{0}}{f_{s}}N_{\mathrm{length}}$ must be a positive integer).

#### 2.1.3. Phase Difference Information in One Period

Here, the phase information corresponding to one spectral line $f_{0}$ is extracted. Let $K_{0}=\frac{f_{0}}{f_{s}}N_{\mathrm{length}}+1$. The phase of the frequency point $f_{0}$ can be found from $F\left(K_{0}\right)$. im means finding the imaginary part, and re the real part. The solution range for the arctangent angle is [−π/2, π/2]:

(5) $$\varphi_{f_{0}}=\arctan[\frac{im(X(K_{0}))}{re(X(K_{0}))}]$$

The arctangent solution output is corrected preliminarily as discussed below:

(6) $$\left\{ \begin{array}{cc} re(X(K_{0}))>0 & \varphi_{f_{0}}=\varphi_{f_{0}}\\ re(X(K_{0}))<0\;im(X(K_{0}))>0 & \varphi_{f_{0}}=\pi +\varphi_{f_{0}}\\ re(X(K_{0}))<0\;im(X(K_{0}))<0 & \varphi_{f_{0}}=\varphi_{f_{0}}-\pi \end{array}\right.$$

Meanwhile, the arctangent angle is found in the [−π, π] range.

#### 2.1.4. Extraction of the Two-Circuit Signal Phase Difference

The two-circuit signal phase difference is given as:

(7) $$\varphi_{ij}=\varphi_{if_{0}}-\varphi_{jf_{0}},$$

where $\phi_{ij}$ and $\phi_{ij}^{\prime }$ are the phase information of the *i*th and *j*th sensor node, respectively, when the P-wave peak frequency is $f_{0}$, and $\varphi_{ij}$ is a phase difference between sensor node *i* and sensor node *j* in a single cycle, where $t_{ij}$ is the corresponding time difference in a single cycle:

(8) $$t_{ij}=\varphi_{ij}/2\pi f_{0}$$

### 2.2. Phase-Difference Calibration Method Based on the First-Break Arrival Time and System Phase Information

**Figure 5:**
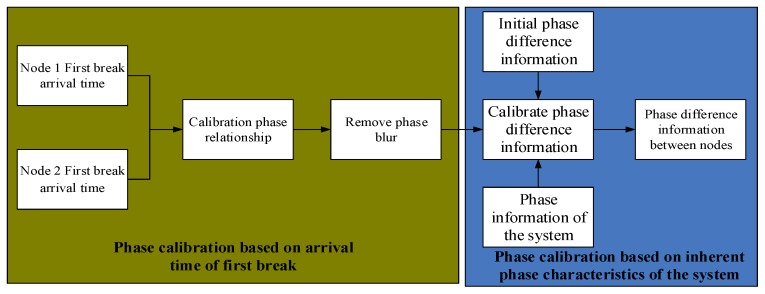
Schematic diagram of phase difference calibration.

The calibration of the phase difference is completed in two steps: the phase difference calibration based on the arrival time of the first break, and the phase difference calibration based on the inherent phase characteristics of the system, as shown in Figure 5.

#### 2.2.1. Phase Calibration Based on Arrival Time of the First-Break

The arrival time of the first break characterizes the sequence of the P-wave reaching each node, that is, the phase relationship. Phase ambiguity is an issue that can be taken care of by addressing the arrival time difference, thereby enhancing the phase difference information [23,24].

Let the time when the first break reaches node *i* (with coordinates of $\left(x_{i},y_{i},z_{i}\right)$) be $t_{i}$ and the time when the first break reaches node *j* ($\left(x_{j},y_{j},z_{j}\right)$) be $t_{j}$. Suppose the wave group travels in the medium at a speed of v. Suppose the highest dominant frequency of the P-wave is $f_{0}$, the phase difference between the two nodes is $\phi_{ij}$ (with $\phi_{ij}\in \left[-\pi ,\pi \right]$), and $\phi_{ij}{}^{\prime }$ is the corrected phase difference. $t_{\phi_{ij}}$ denotes the time difference corresponding to the corrected phase difference. According to the wave theory, we have Equations (8) and (9), where *N* (a major correction variable) is the number of times the period of the frequency component is repeated; the time-difference information corresponding to nodes *i* and *j* is given using Equation (11):

(9) $$\phi_{ij}^{\prime }=\phi_{ij}+2\pi N$$

(10) $$t_{\phi_{ij}}=\phi_{ij}^{\prime }/2\pi f_{0}$$

(11) $$\Delta t_{ij}=t_{i}-t_{j}$$

**Figure 6:**
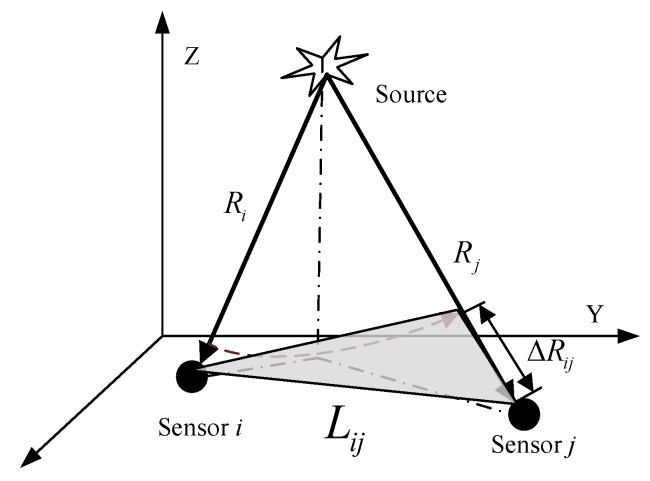
Schematic diagram of sensor geometry constraints.

According to the sensor array geometry and wave field propagation theory, as shown in Figure 6, the constraints of the time-difference information can be obtained.

Let the linear distance between the two sensor nodes be $L_{ij}$, the difference between the distances traveled by the P-wave from the source to nodes *i* and *j* be $\Delta R_{ij}$, the corresponding time difference be $t_{\phi_{ij}}$, the time difference between the first-break of the P-wave reaching the two nodes be $\Delta t_{ij}$, and the travel speed of the P-wave be *v*.

As seen in Figure 6, $L_{ij}$ is the longest side of the gray triangle; therefore, at the same travel speed *v*, $t_{\phi_{ij}}<t_{L_{ij}}$. Considering the number of period repetitions of the frequency component N≥0, we get $\Delta t_{ij}\leq \Delta t_{\phi_{ij}}$. Hence, Equation (12) is satisfied:

(12) $$\Delta t_{ij}\leq t_{\phi_{ij}}<\frac{\sqrt{{\left(x_{i}-x_{j}\right)}^{2}+{\left(y_{i}-y_{j}\right)}^{2}+{\left(z_{i}-z_{j}\right)}^{2}}}{v}.$$

In the middle:

(13) $$t_{L_{ij}}=\frac{L_{ij}}{v}=\frac{\sqrt{{\left(x_{i}-x_{j}\right)}^{2}+{\left(y_{i}-y_{j}\right)}^{2}+{\left(z_{i}-z_{j}\right)}^{2}}}{v},$$

(14) $$\frac{2\pi f_{0}(t_{i}-t_{j})-\phi_{ij}}{2\pi }\leq N<f_{0}.\frac{\sqrt{{\left(x_{i}-x_{j}\right)}^{2}+{\left(y_{i}-y_{j}\right)}^{2}+{\left(z_{i}-z_{j}\right)}^{2}}}{v}-\frac{\phi_{ij}}{2\pi }.$$

From Equations (12)–(14), the constraint of *N* can be found. Also, with the first break arrival time known for the two nodes, the phase relationship of the P-wave’s highest dominant frequency between the two nodes can be determined:

(15) $$\left\{ \begin{array}{ll} \Delta t_{ij}>0 & \left(\mathrm{Node}\;i\;\mathrm{leads}\;\mathrm{node}\;j\right)\\ \Delta t_{ij}<0 & \left(\mathrm{Node}\;i\;\mathrm{lags}\;\mathrm{node}\;j\right) \end{array}\right.$$

#### 2.2.2. Secondary Phase Calibration Based on the System Phase Characteristics

In a wireless sensor network, the phase–frequency characteristics of each node are not completely identical. Therefore, there inevitably exists some systematic errors in the phase difference between the nodes. To improve the phase difference measurement accuracy, a calibration is made based on the phase frequency characteristics of the sensors [25,26].

The phase difference $\phi_{ij}$ is corrected using the arrival time recorded by the two nodes to get $\phi_{ij}{}^{\prime }$. Let $\phi_{i}$ be the intrinsic phase value of the *i*th node at the dominant frequency $f_{0}$, $\phi_{j}$ be the intrinsic phase value of the *j*th node at the dominant frequency $f_{0}$, and $\phi_{ij}^{\prime \prime }$ be the phase difference information after the secondary correction:

(16) $$\phi_{ij}^{\prime \prime }=\{\begin{array}{ll} \phi_{ij}^{\prime }-(\phi_{i}-\phi_{j}) & \phi_{i}>\phi_{j}\\ \phi_{ij}^{\prime }+(\phi_{i}-\phi_{j}) & \phi_{i}<\phi_{j} \end{array}.$$

Thus, as well as solving the phase ambiguity problem, the two calibration operations—the phase calibration based on the arrival time of the first break and the secondary phase calibration based on the phase characteristics of the system—have corrected the phase error of the system.

From Equation (17), we can find the time difference, where $t_{\phi_{ij}}$ is the corrected time difference, $t_{\phi_{ij}}$ is the dominant frequency, and $\phi_{ij}^{\prime \prime }$ is the corrected phase difference:

(17) $$t_{\phi_{ij}}=\phi_{ij}^{\prime \prime }/2\pi f_{0}.$$

## 3. Hardware Implementation of the FPGA-Based Time-Difference Measurement Circuit

This system was based on a Zynq series FPGA. It combined a WiFi, a multi-channel Analog-to-Digital Converter (ADC), a Flash Memory, and a Double Data Rate 3 (DDR3) to form a hardware system for time-difference measurement. The DDR3 acted as the extended memory of the FPGA. The ADC and the Flash together made a multi-channel data acquisition and storage module, while the WiFi worked as a wireless data transceiver module [27].

**Figure 7:**
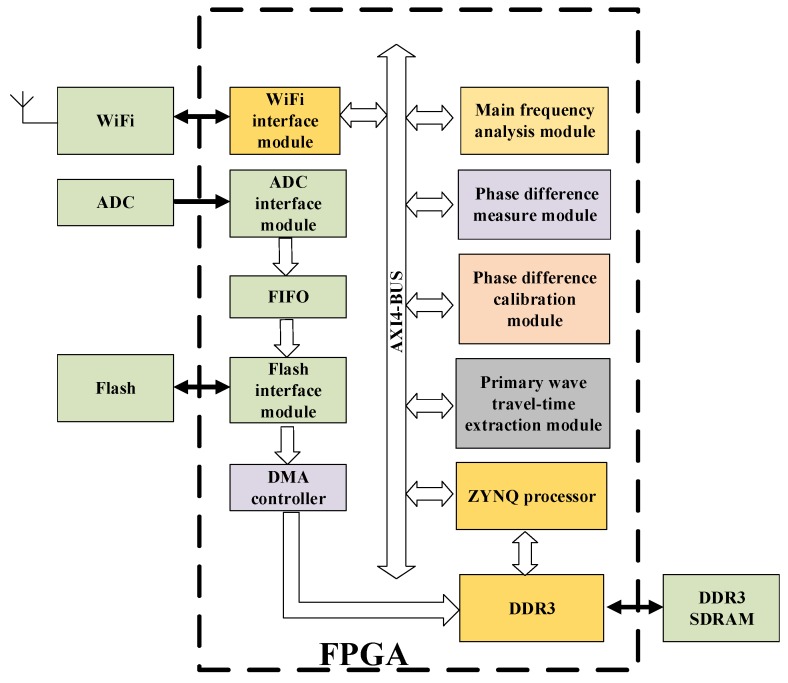
Overall schematic diagram of FPGA top layer design.

In the FPGA platform, the overall design idea of the software was as shown in Figure 7. It included a wireless Wireless Fidelity (WiFi)driver, an ADC driver, an First Input First Output-Intellectual Property (FIFO-IP) core, a Flash driver, a main frequency measurement module, a first-break arrival time extraction module, a phase difference measurement module, a phase difference calibration module, a DDR3 controller, and a Zynq processor. The main frequency measurement module, the first-break arrival time extraction module, the phase difference measurement module, the phase difference calibration module, the DDR3 controller were connected to the Zynq processor through the Advanced eXtensible Interface 4.0 (AXI4) bus [28]. The Flash transmitted data to the DDR3 in the Direct Memory Access (DMA) mode.

With this system, the time-difference measurement was done in three steps: (1) acquire, via the ADC, FIFO, and Flash, the completely stored blasting vibration data; (2) transfer, by means of the DMA, the data to the large-capacity DDR3; and (3) with the DDR3 as memory and the remaining time-difference measurement modules as the peripheral, evaluate the time difference by finding the addresses of the peripheral devices through the AXI4 bus.

**Figure 8:**
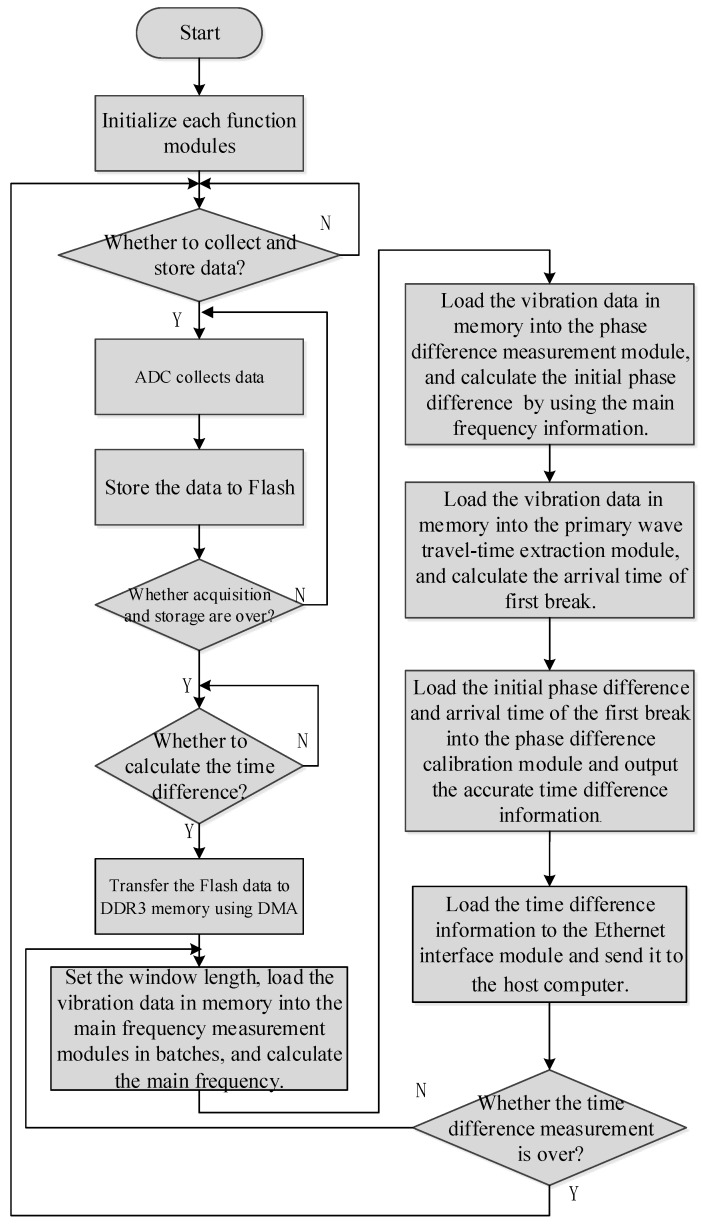
ARM control flowchart.

Figure 8 shows the flow of how the Zynq’s internal ARM controlled the peripheral modules, where the ARM controlled each of the modules in a sequential execution mode. The specific control flow was as follows:

S1. Initialize each peripheral module and the ARM.

S2. Determine whether to start acquiring the blasting data. Once the acquisition instruction is received, the ADC, FIFO, and Flash cooperate to save multiple sensor signals in the Flash. When the acquisition and storage ends, the flag of the end of the acquisition is fed back to the ARM.

S3. Wait for the instruction of time-difference calculation. After receiving the instruction, the sensor data stored in the Flash is transmitted to the DDR3 by means of the DMA.

S4. After the data loading is completed in the DDR3, the phase difference measurement module is enabled. The module finds the master frequency position using FFT analysis and a peak comparison, and calculates the initial phase difference information in the frequency domain by calling Coordinate Rotation Digital Computer (CORDIC) IP, and then sends the information back to ARM.

S5. Enable the first-break arrival time extraction module and return the arrival time information of different sensors.

S6. Input the initial phase difference information and the first-break arrival time information to the phase difference calibration module, and output accurate time-difference information.

S7. Send the time-difference information to the host computer through the WiFi interface.

S8. At the end of the time-difference measurement, wait for the host computer to give instruction.

### 3.1. Design of the Master Frequency Measurement Module

**Figure 9:**
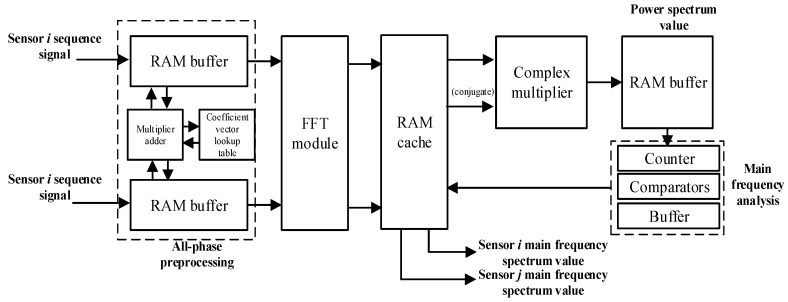
Master frequency measurement circuit block diagram.

As the first step, the full-phase preprocessing coefficient vector was calculated and quantized in Matlab R2014a, and the coefficient vector was then exported to generate a ROM lookup table [29]. The input signals of the two circuits were stored in the RAM buffer, where the necessary multiplication and addition operations were performed according to the coefficient vector lookup table, and the full-phase preprocessing and buffering of the two-circuit signals were completed [30]. Second, the first-circuit signal, after being preprocessed, received a Fourier transform in the FFT module and was then cached. The output signal from the FFT module, along with its conjugate, was fed to the complex multiplier to produce the corresponding power spectrum value, which was then cached. Then, from the cached power spectrum value, the master frequency was determined by means of a peak comparison. The second circuit vibration signal was processed in the same way to determine the master frequency common to the two signals. The actual implementation circuit is shown in Figure 9.

### 3.2. First-Break Arrival Time Circuit Module

**Figure 10:**
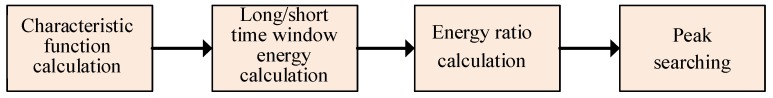
Flowchart of arrival time extraction.

The system used a negative delay, which had a depth of 0.5 s, to trigger the acquisition module. Therefore, upon the end of a storage operation, the first second of each data set (4096 points of data) was read, where the arrival time was extracted. The first-break arrival time extraction module chose a time window length of 32 ms (128 points). There were four steps, which were the feature function calculation module, the long and short time window energy value calculation module, the energy ratio calculation module, and the peak search module, as shown in Figure 10. 

#### 3.2.1. Feature Function Calculation Module

The functions for inputting x(i) and outputting CF(i) were enabled. The feature function CF(i) is expressed as:

(18) $$CF(i)=\left|x^{2}(i)-x(i)x(i-1)\right|.$$

This module consisted of two multipliers and two subtractors. The two multipliers were each responsible for computing x(i)×x(i) and x(i)×x(i−1), and they gave their results as P1 and P2, respectively. Two subtractors each computed P1 − P2 and P2 − P1, with the positive of the two being the output.

**Figure 11:**
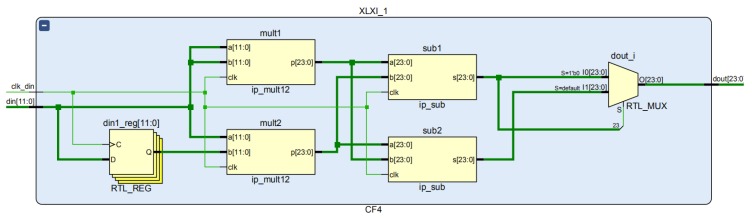
RTL diagram of the feature function module.

Figure 11 shows how the feature function calculation module implemented the Register Transfer Level (RTL).

#### 3.2.2. The Long and Short Time Window Energy Calculation Module

Two registers were used to store the *STA* and *LTA* energy values. The probable maximum value of the time window energy values ought to be estimated to determine the bit width of the registers, lest there might be data overflow over the course of the calculation.

Since the length of the short window was 128 points, and the length of the long window was four times the length of the short window, i.e., 512 points, the data was 24 bit, such that the cumulative value of the long window was $512\times 2^{24}=2^{33}$, which exceeded 32 bits. This would consume an excessive amount of hardware resources, which was not conducive to the implementation of the ensuing division operation. Therefore, the previous calculation result of the feature function sequence was truncated, discarding the last 7 bits such that 18-bit significant data was retained. In this way, the *STA* and *LTA* calculation values were smaller than $4\times 10^{6}$; that is, they could be represented by a set of 24-bit registers.

The calculation of the *STA* and the *LTA* values was done using a recursive formula given by Equations (19) and (20):

(19) STA(n)=STA(n−1)+CF(n)−CF(n−Nsta),

(20) LTA(n)=LTA(n−1)+CF(n−Nsta)−CF(n−Nsta−Nlta).

**Figure 12:**
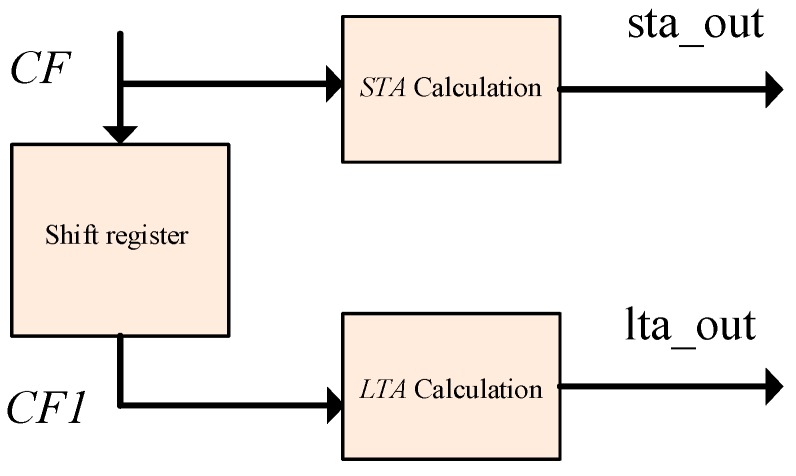
Long- and short-term average (*LTA*/*STA*) window calculation design.

It was necessary to generate a shift register to cache up the 128-point feature function sequence. At both ends of the shift register were the data sources for computing the *STA* and the *LTA* values. The block diagram of the program design is shown in Figure 12. The calculation process involved addition, subtraction, and data shift registering. As before, a state machine was used for control. A data clock controlled the data shift register, and the system clock drove the state machine. The specific process will not be described here. Nevertheless, should there occur data overflow during the calculation process, i.e., the “carry” output signal of the adder was high, this might have been due to loose sensor wiring or sensor damage.

#### 3.2.3. The Division Module

In calculating the ratio between the long and short window energy values, it was necessary to perform the division operation. In the calculation process, the divisor *LTA* may have a zero value, in which case, the result of the division calculation becomes unpredictable. To preclude such a situation, before each division operation, a value of 1 was first added to the data input by the divider, which ensured the correctness of the output result. This step was actually completed in step (2), when the final output of the long and short time window energy value calculation results were completed, as shown in Equation (21):

(21) $$R=\frac{STA+1}{LTA+1}.$$

A distortion-free integer division operation was possible using the Xilinx divider IP core. The quotient and remainder of the divisor calculation were integers (the result of a modulo operation), the data input supported a 2- to 32-bit width, and the divider was of a stream mode by design, allowing for a complete division operation in one clock cycle.

**Figure 13:**
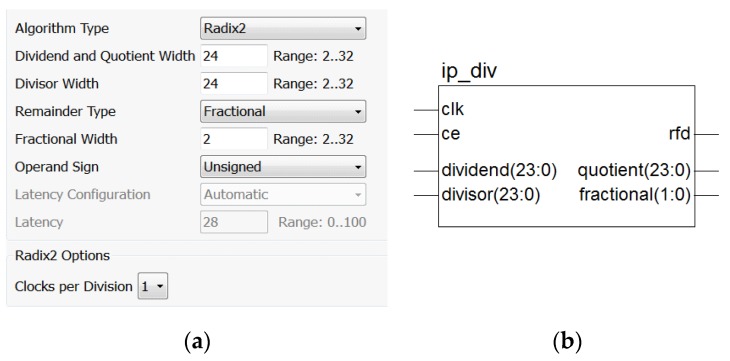
Operation diagram of the divider: (**a**) divider module configuration diagram and (**b**) divider module interface diagram.

From the input of the dividend and the divisor to the output of the operation result, there was a delay of K clock cycles, with the value of K being related to the data bit width and the configuration mode of the divider. Here, the divider was configured according to a fully synchronized design of a single clock, with both the divisor and the dividend being 24-bit unsigned data inputs. Since the remainder was not relevant here, its width was set to 2, the minimum width. The key configuration steps and the module schematic are shown in Figure 13.

In the interface shown in Figure 13, “ce” represents clock enabled and active high, “dividend” is the input dividend, “divisor” is the input divisor, the output “quotient” is the quotient, and “fractional” is the remainder. “rfd” is a data ready signal. As the number of the division operation clock was 1, the divider sampled the input data on each rising edge of the clock.

#### 3.2.4. The Peak Value Search Module

**Figure 14:**
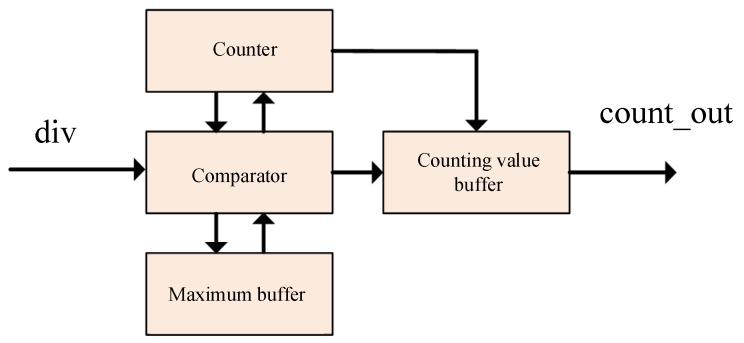
Peak search module design.

The peak search module was used to find the time at which the energy corresponded to the maximum value. As with the implementation design, there was a need for a counter to record the current data location, a data buffer for storing the current maximum value, a location buffer for caching the current maximum location, and a comparator for comparing the current input with the current maximum. The location buffer and data buffer were updated if the current input value was greater than the cache maximum. The design of the peak finder is shown in Figure 14.

**Figure 15:**
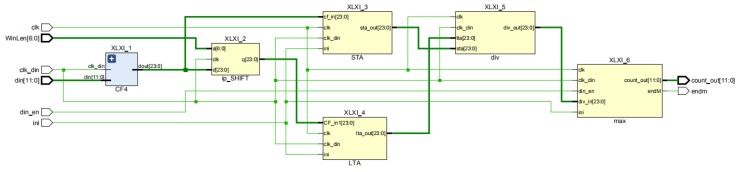
RTL top-level schematic of the arrival time extraction module.

The overall RTL schematic diagram of the arrival time extraction module is shown in Figure 15.

### 3.3. Design of the Phase-Difference Measurement and Calibration Circuit Module

**Figure 16:**
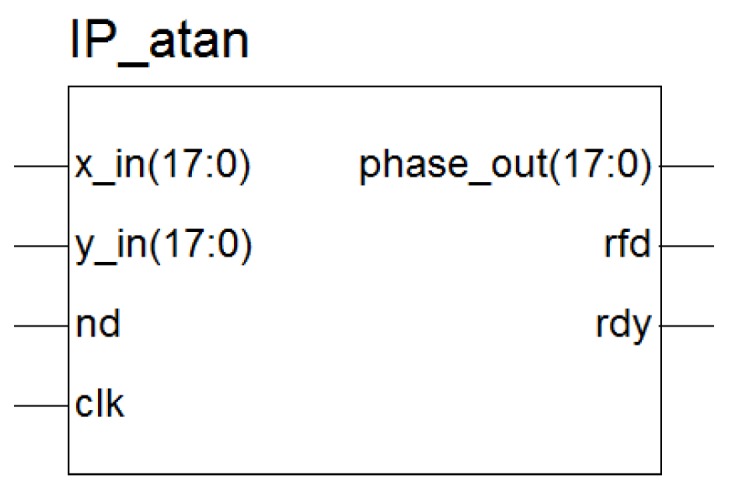
Interface diagram of the phase calculation module.

First, the CORDIC IP core generated an arctangent phase calculation module, into which the spectral values of the two-circuit signals were fed as input data. The schematic diagram of the phase evaluation module based on CORDIC is shown in Figure 16. 

“x_in” is the real part of the input data; “y_in” is the imaginary part of the input data; and “phase_out” is the output phase angle, in radians. x_in, y_in, and phase_out are represented as binary complements. ”nd” is the new sampling mark signal for the input port. “rfd” indicates that the module is ready to sample new data. The “rdy” indicator signal means that there was new valid data at the data output port.

**Figure 17:**
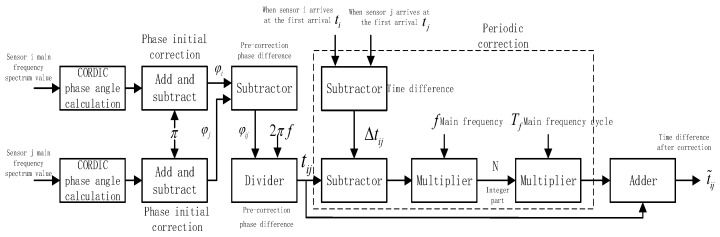
Block diagram of the phase-difference measurement and calibration circuit design.

Depending on the positive or negative sign of the real and imaginary parts of the spectral values, the two phase angles were first corrected by the adder-subtractor to give $\varphi_{i}$ and $\varphi_{j}$. Second, the subtractor evaluated $\varphi_{j}-\varphi_{i}$, and the result was then used as the dividend for the divider, with the divisor being 2πf, and the pre-correction time difference $t_{ij}$ was found. From the first-break arrival time circuit module, the arrival time of the two circuit signals—$t_{i}$ and $t_{j}$—were found, and their difference was $\Delta t_{ij}=t_{i}-t_{j}$. Again, $\Delta t_{ij}-t_{ij}$ was evaluated by the subtractor, whose value was then multiplied by the master frequency (i.e., divided by the period of the master frequency), and the integer part (*N*) of the product was the phase fuzzy period number. Finally, the multiplier evaluated (the period length of the master frequency) × (the phase fuzzy period number, *N*), with the result being added to the pre-correction time difference $t_{ij}$ to produce the corrected time difference ${\tilde{t}}_{ij}$. The actual implementation circuit is shown in Figure 17.

### 3.4. FPGA-Based Circuit Implementation

**Figure 18:**
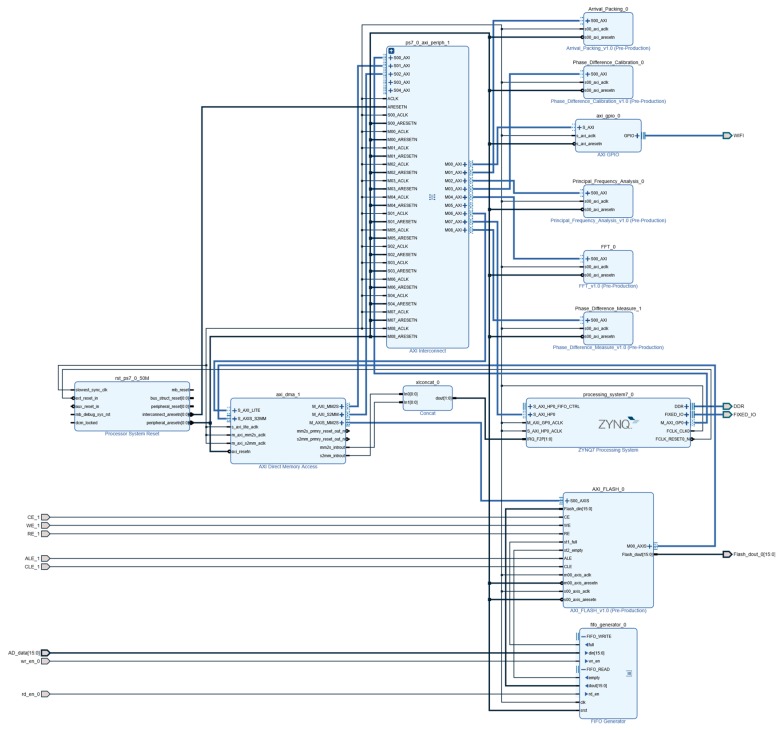
Detailed schematic of the top-level design.

The extraction circuit of the time-difference information was designed based on FPGA, the specific design process is shown in Figure 18. First, in the Vivado platform, program codes were developed for the ADC, the master frequency measurement, the first-break arrival time extraction, and the phase difference measurement and calibration modules. Furthermore, the corresponding AXI4-type IP core was generated. Second, a block design was created; the generated IP cores were loaded one by one into the diagram; and IP cores, such as the Zynq7 processing system, DMA, and FIFO, were included. Then, the AXI interconnect module (i.e., AXI interconnection matrix) was used to connect each AXI4 IP core to the Zynq7 processing system, realizing the function of data interacting among the multiple modules [31]. After that, the relevant input and output interfaces were exported to complete the programmable logic part (PL). Finally, the ARM processing system was developed in the Software Development Kit (SDK) platform, mainly covering the workflow of each module, and the matching between data interfaces (such as the time-difference information) and the wireless transmission module.

This project, by means of the DMA, completed the data interaction between the Flash and the DDR3. The DMA interrupt signal was connected to the xlconcat component, inputting the interrupt signal into the Zynq IP core. When the DMA operation was completed, an end interrupt was issued to the CPU, informing the CPU to read the memory data for the next time-difference measurement.

## 4. Algorithm Simulation and Hardware Simulation

### 4.1. Simulation Validation of Spectral-Decomposition-Based Phase-Difference Measurement

**Figure 19:**
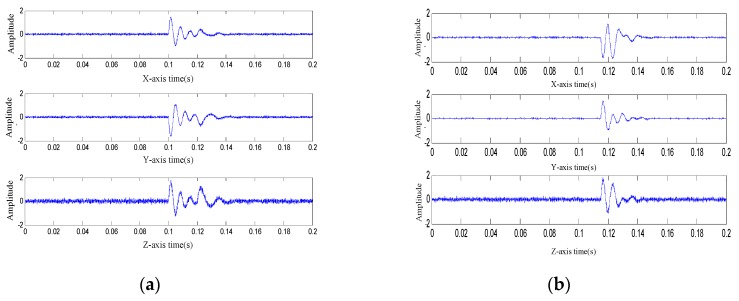
Simulated blasting vibration signals: (**a**) diagram of three component signals of the blasting vibration for node 1 and (**b**) diagram of three component signals of the blasting vibration for node 2.

To evaluate the feasibility of the proposed method, two sets of blasting vibration waves were generated artificially. The signal sampling rate was set at 20 kHz and the sampling time was set to 0.6 s. In the case of the first blasting vibration wave, the arrival time of the first break was 0.1 s, the frequency was 160 Hz, and the attenuation factor was $e^{-i/160}$ (i=0,1,⋯,n). The angle the longitudinal wave beam model formed with the x-axis was π/6, or π/3 with the y-axis, or π/3 with the z-axis. The shear wave was generated at 0.12 s with a frequency of 80 Hz and an attenuation factor of $e^{-i/160}$ (i=0,1,⋯,n), and the angle between the shear-wave beam model and the x-axis, the y-axis, and the z-axis was π/3, π/3, and π/6 respectively. For the second wave group, the arrival time of the first break was at 0.115 s, the longitudinal wave frequency was 160 Hz, and the attenuation factor was $e^{-i/150}$ (i=0,1,⋯,n); the angle between the longitudinal wave beam model and the x-axis, y-axis, and z-axis was −π/6, π/3, and π/6, respectively. The shear waves started their linear superimposition at 0.135 s with a frequency of 80 Hz and an attenuation factor of $e^{-i/160}$ (i=0,1,⋯,n), and the shear wave beam model formed an angle of −π/6, π/6, and π/3 with the x-axis, y-axis, and z-axis, respectively. The initial phase difference between the longitudinal wave of the two wave groups was 14.13 rad, and the noise source was Gaussian white noise with a signal-to-noise ratio(SNR) of 35 dB. The waveform of the two sensor nodes are shown in Figure 19.

**Figure 20:**
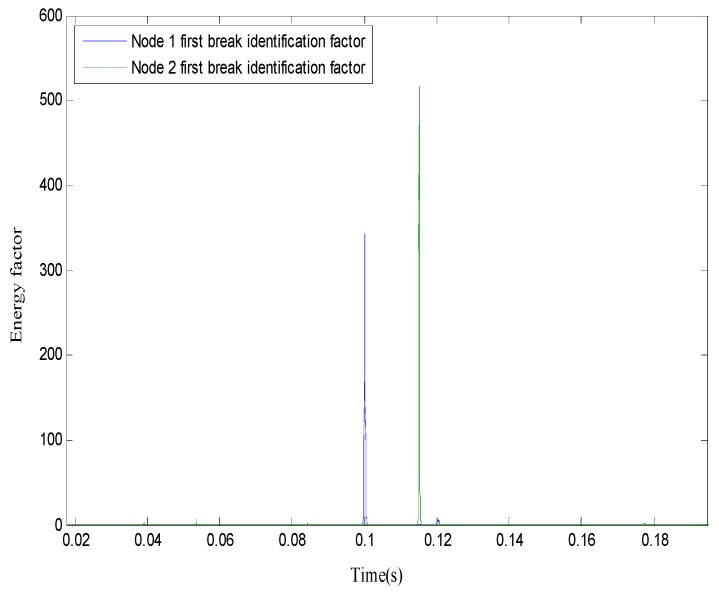
Identification factor graph.

The arrival time of the first break of the two nodes was extracted using the STA/LTA algorithm, as shown in Figure 20.

The peak factor and the corresponding arrival time of the first break were extracted according to the maximum peak principle. The results are given in Table 1, which show that the time difference between node 1 and node 2 was 0.01510 s.

**Figure 21:**
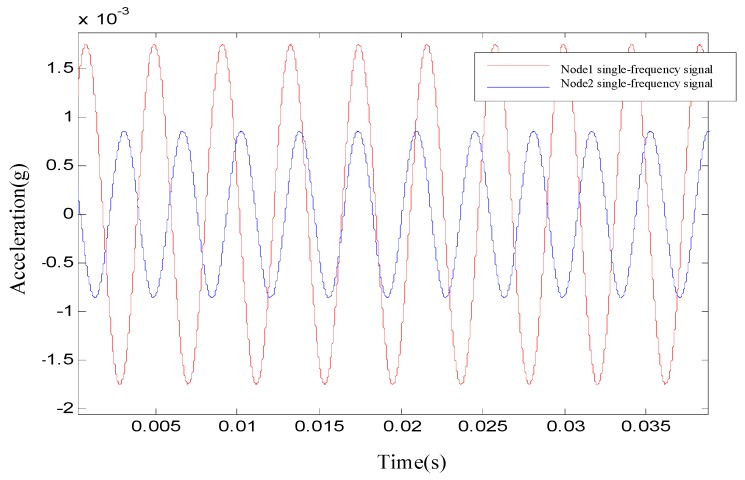
Time domain waveform of two nodes at the dominant frequency.

After the time–frequency analysis, the 160 Hz signal was extracted for phase difference determination, as shown in Figure 21.

After the time–frequency analysis, the 160 Hz signal was extracted for phase difference determination. Equation (5) gave the phase difference between the two nodes as $\phi_{ij}$= 1.574364 rad. With the first break arrival times, which were already known, Equation (10) gave the waveform repetition period as *N* = 2 and the corrected phase difference as $\phi_{ij}{}^{\prime }$ = 14.133436 rad. The corresponding time difference was 0.0150045 s. Table 2 presents more values.

Given Table 1 and Table 2, and using a phase difference determination, the time-difference measurement accuracy was improved from 0.6% to 0.3% The temporal resolution was raised from 5 × 10^−5^ to 5 × 10^−7^.

**Figure 22:**
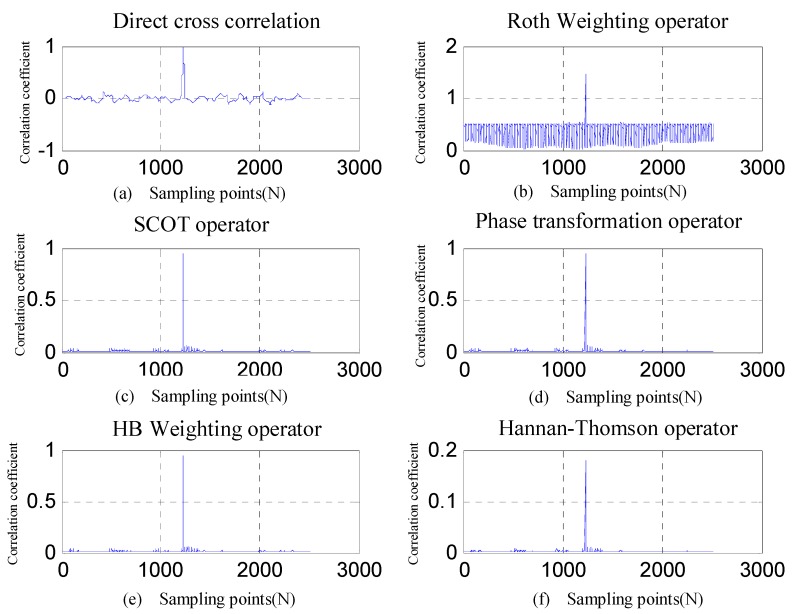
Time-difference measurement results based on generalized correlation methods: (**a**) direct cross-correlation, (**b**) Roth weighting operator, (**c**) smoothed correlation transform (SCOT) operator, (**d**) phase transformation (PHAT) operator, (**e**) HB weighting operator, and (**f**) Hannan–Thomson operator.

To further verify the feasibility of the proposed method, the time difference was determined using six generalized cross-correlation methods (direct cross-correlation, Roth impulse shock response, smoothed correlation transform (SCOT), phase transformation (PHAT), HB weighting, and maximum likelihood (Hannan–Thomson)). The results are shown in Figure 22, where the abscissa represents the number of sampling points and the ordinate represents the normalized spectral peak.

The generalized correlation spectral peaks in Figure 22 were searched, and the number of sampling points corresponding to the peaks was converted into time-difference information, as given in Table 3.

It can be seen from Table 3 that the time-difference information given by these generalized cross-correlation methods varied greatly, mainly because the shock wave generated by the near field of the blasting was characterized by slow peaking, a complex waveform, and multi-wave aliasing. The first pulse peak point was not necessarily the highest in the full waveform. For this reason, the detected peak was tainted by misjudgment. Although the smooth correlation transform (SCOT), the phase transformation (PHAT), the HB weighting, and the maximum likelihood (Hannan–Thomson) methods gave obvious peaks, a great discrepancy existed in the time-difference information they gave.

As can be seen in Table 4 and Table 5, since the first break arrival time was extracted based on the initial take-off point of the shock wave, there was no possible misjudgment of the time difference, and the performance was therefore better than the time-difference extraction methods based on a generalized cross-correlation. The proposed phase measurement method was an improvement on the extraction method based on the first break arrival time and improved the resolution of the time difference by two orders of magnitude. It significantly improved the accuracy of the time-difference measurement, and thus afforded a reliable basis for subsequent high-precision source localizations.

### 4.2. FPGA-Based Hardware Simulation Validation

#### 4.2.1. Spectrum Analysis Module

Signals with a frequency of 100 Hz, 200 Hz, 300 Hz, and 400 Hz were generated using Matlab, with each signal being sampled at a rate of 1 kHz for a duration of 2 s. After being superimposed, the four signals were subjected to all-phase preprocessing. The preprocessed signal was quantized, to be used as an input signal to the FFT module.

**Figure 23:**
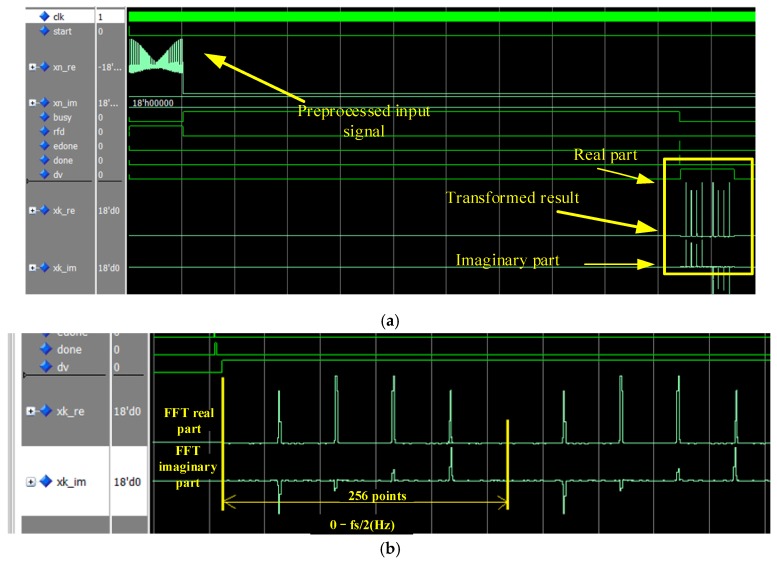
Waveform simulation diagram of the spectral analysis module: (**a**) all-phase FFT module test overall diagram and (**b**) all-phase FFT module test partial magnified diagram.

Figure 23 shows the calculation result of the FFT module. The control input signal “start” was one clock cycle at a high-level, at the end of which, “rfd” will go the high level, indicating that the FFT module was ready to receive data. The xn_re and xn_im sequences were simultaneously input into the next 256 clock cycles. When the data were all input, “rfd” went to the low level, and “busy”—suggesting the FFT was calculating—went to the high level. At the beginning of the next clock cycle, in which the pulse signal “edone” occurred, or at the beginning of the clock cycle in which the pulse signal “done” occurred, xk_re and xk_im began to output the conversion result. As shown in Figure 23, the original input signal was four cosine signals superimposed together, and the output signal after the FFT transformation reflected four sets of conjugate symmetric lines.

#### 4.2.2. Phase Measurement Module

**Figure 24:**
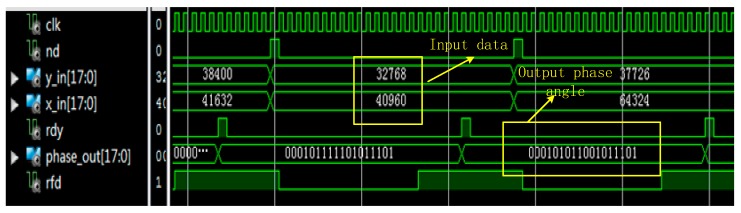
Simulation waveform of the phase measurement module.

“x_in” was the real part of the input data, “y_in” was the imaginary part of the input data, and “phase_out” was the output phase angle in radians. x_in, y_in, and phase_out were represented as binary complements. “nd” was the new sampling mark signal for the input port. “rfd” indicated that the module was ready to sample new data. The “rdy” indicator signal meant that there were new valid data samples at the data output port. Figure 24 shows the simulation results of the arctangent operation module. 

When “nd” was at a high level, the module sampled new data while the clock was on the rising edge. The “rdy” output was at a high level during the first clock cycle in which valid data appeared on the output port. As shown, with the input y_in = 32,768 and x_in = 40,960, the output phase_out was “000101011001011101”; the first 3 bits of the phase_out output represented the integer part, and the last 15 bits represented the fractional part. After the conversion, we got phase_out = 0.6747, which was in line with the Matlab value.

#### 4.2.3. DMA Data Flow Test

The system included the FIFO, master frequency measurement, first break arrival time extraction, time-difference measurement, and calibration modules or circuits, which were connected to the Zynq core through the AXI4 bus. In the above modules, the data acquisition module, composed of the ADC, FIFO, and Flash, and the circuit module DMA connecting the Flash to the DDR3 all had high requirements regarding data flow.

The system set the ADC sampling rate to 100 Ksps, having a quantization bit number of 16 and a sampling time of 10 s. The Flash stored the data in pages, the size of each page being 2 KB. Therefore, the FIFO was set to 4 KB, and the Flash was controlled using a half-full signal to store data. There was no data loss, since the speed at which the ADC wrote to the FIFO was 1.6 Mbps and the speed at which the Flash read data from the FIFO was 32 Mbps.

For the time-difference measurement, the sensor data stored in the Flash were transmitted to the memory through the DMA. Therefore, this approach needed to be verified for its reliability. In this paper, the test was done in a PS–DMA–PL–DMA–PS loop architecture (PS—processing system, PL—programmable logic). This method verified whether the data written to the peripheral via the DMA were consistent with the data written from the peripheral to the memory via the DMA.

**Figure 25:**
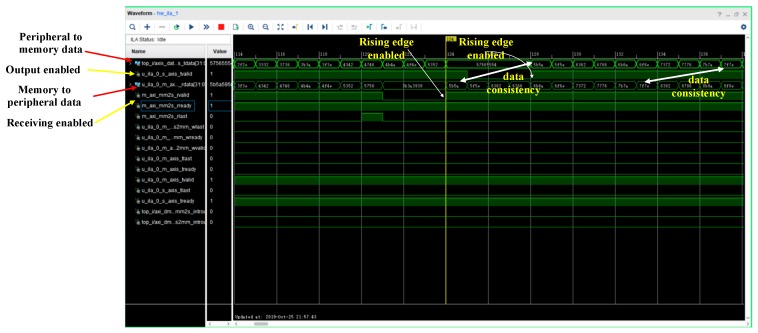
DMA data flow test chart.

The signal was generated on the Vivado platform using simple_poll, an SDK development routine. In the test phase, Interactive Logic Array(ILA) was opened in the debug mode, and the trigger condition of the Memory Map to Stream (MM2s) was set to a rising edge. The waveform, after triggering, was as shown below. As can be seen from Figure 25, the data written to the peripheral via the DMA were consistent with the data written from the peripheral via the DMA to the memory, indicating that the data stream was transmitted without error.

## 5. Field Testing

**Figure 26:**
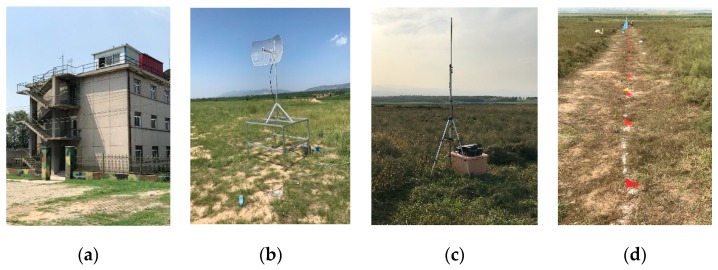
Field layout of the control distance of wireless sensor nodes: (**a**) main control station, (**b**) field transmitting antenna, (**c**) wireless AP, and (**d**) test site node layout.

As a way to test the real-time computing performance of the system, a self-developed wireless sensor network was constructed that consisted of 64 sets of sensor nodes, a wireless Access Point(AP), and wireless bridges for subsurface vibration source localization. The test site, which was 8 km away from the control main station, was loaded with 10 kg Trinitrotoluene(TNT) to create an artificial vibration source. Following the quake, two solutions—the terminal data post-processing and on-chip real-time processing—were used to evaluate the accuracy of the time-difference information extraction, speed determination, and source location. The test site layout is shown in Figure 26.

**Figure 27:**
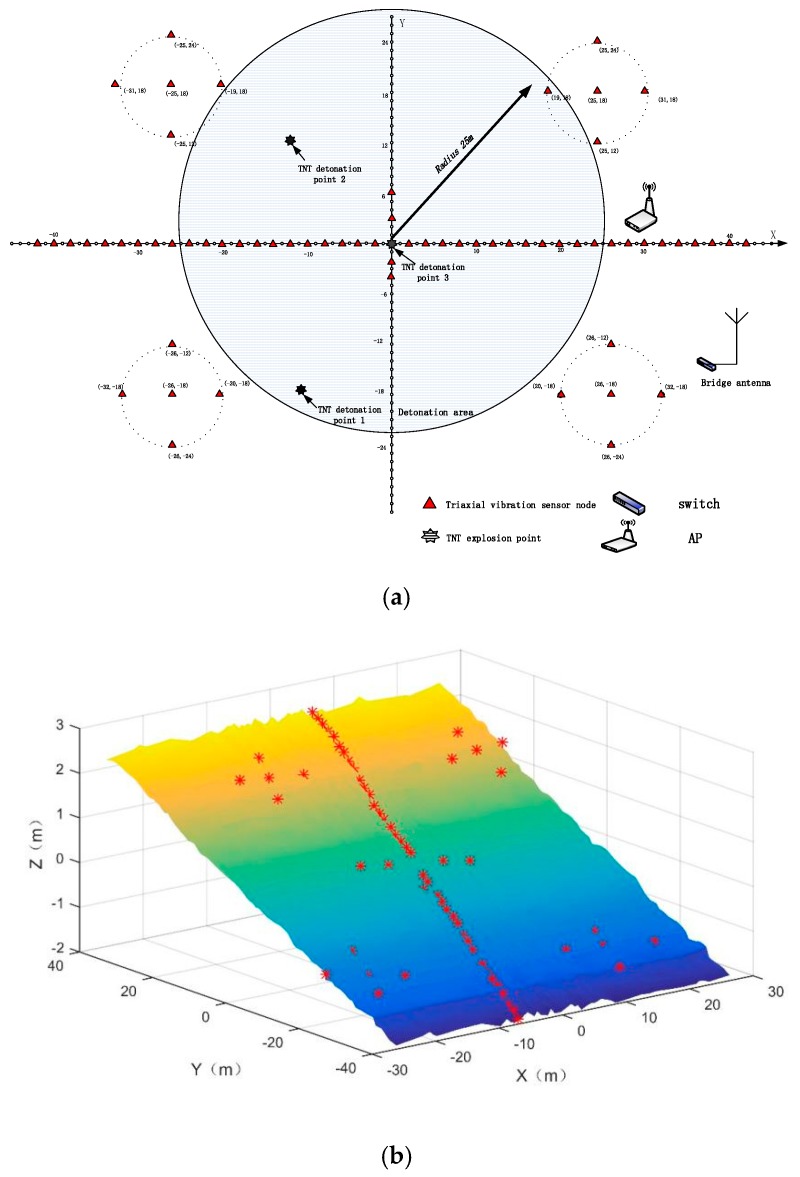
Layout of the sensors: (**a**) plane layout of the sensors and (**b**) actual position coordinates of the sensors and topographic map.

The 64 sensor nodes were arranged in linear arrays and five-point cross arrays, as shown in Figure 27. The elevation was not a concern in the layout. The location of the sensors was determined using the high-precision Beidou and then recorded.

**Figure 28:**
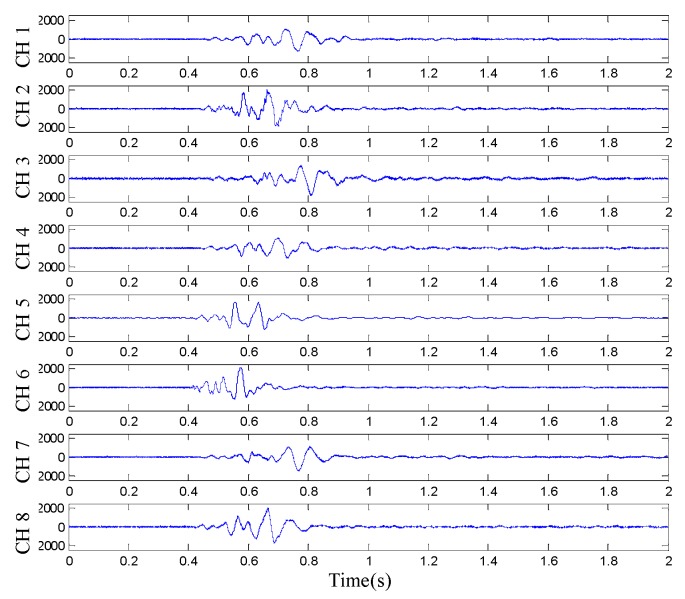
Read data waveform.

The sampling rate of the multi-channel data acquisition system was set to 100 kHz and its sampling time was set to 10 s. After the end of the test, the acquired data and the time-difference information determined by the nodes were transmitted back. The Figure 28 shows the waveform of the raw vibration signals transmitted back.

Using the No. 4 sensor as the reference, the time-difference information and positioning results that were obtained are tabulated in Table 4.

The second row in Table 4 is the time-difference information extracted by the system hardware. Since the ADC sampling rate was 100 kHz, the minimum resolution of the time difference was 10 μs. It took 5 ms from the beginning of the time-difference calculation to the transmission of this information to the host computer. If the time difference was calculated by the traditional upper computer, the sensor array data had to be transmitted back to the upper computer, involving a wireless sensor network transmission speed of 200 KB/s. Therefore, with the 64 sensor points acquiring data at a sampling rate of 100 kHz for 10 s, the data volume would be 128 MB, which would take 640 s to transmit; if the software operation time of the computer personnel is considered, the total time would be 750 s. On the other hand, the eight-channel arrival time information and the relative time-difference information processed by the hardware merely took up 128 bytes, which took a negligible time for transmission; both the simulation and the actual testing showed that the node data processing took less than 5 ms. As can be seen, finding the vibration source location when the wireless sensor nodes were arranged at a higher density allowed for the proposed system to be able to shorten the positioning time remarkably.

In a real test, the real-time time-difference information between the nodes was an unknown. Therefore, the measurement accuracy of the time difference was evaluated from the source positioning results. A Geiger source location model was built on the measured time-difference information to produce the location results shown in Table 5.

It can be seen from Table 5 that the time-difference information acquired using both methods produced a similar location accuracy, differing by 0.02 m, or an inconsistency of 3%, as evaluated using the same source localization algorithm. For shallow underground positioning, using engineering standards, the two methods can be considered to be consistent in terms of their positioning effect. In other words, the proposed time-difference measurement method was able to achieve the effect of a host computer. Moreover, the proposed method worked much quicker than the traditional upper computer post-processing method. Therefore, in the field of fast and real-time source localization, the proposed method offers some engineering application value.

## 6. Conclusions

To address problems in TDOA-based shallow subsurface vibration source location, such as low time-difference measurement accuracy and poor real-time performance, this paper proposed a new method for time-difference measurement that was based on phase-difference information. Furthermore, actual circuits were implemented using FPGA. The method determined the direct P-wave’s dominant frequency common to the nodes through Stockwell-transform-based time–frequency analysis, and the phase difference information between the nodes was extracted through a cross-correlation and spectral decomposition algorithm. The first-break arrival time was used to remove phase ambiguity. 

Verification was done using both numerical simulations and experiments, and suggested that compared with the generalized cross-correlation-based time-difference measurement methods, the proposed method produced higher time-difference resolution and accuracy. Compared with the traditional host computer post-position positioning method, the proposed method was significantly quicker. This paper provides a new method for improving positioning speed and accuracy, affording certain engineering application value in the field of real-time underground source localization.

## Figures and Tables

**Table 1:** The first-break arrival wave of the two nodes.

Algorithm	Peak Energy of the First-Break Picking Factor	Measured Arrival Time of First Break (s)	Theoretical Arrival Time of First Break (s)	Time Difference (s)
Node 1	320	0.10005	0.10000	0.01510
Node 2	505	0.11515	0.11500	

**Table 2:** Parameter values determined based on the phase difference information.

Parameter	First-Break Time Information (s)		Extraction Frequency (Hz)	Phase of One Cycle (rad)	Phase Ambiguity Period
Measured value	0.10005	0.11515	160	1.57436	N = 2
**Parameter**	**Running Time (s)**		**Measured Time Difference (s)**	**Theoretical Time Difference (s)**	**Absolute Error**
Measured value	0.778571		0.0150045	0.01500	0.000045

**Table 3:** Time-difference measurement results based on the generalized correlation method (unit: s).

Algorithm	Cross-Correlation	Roth Operator	SCOT Operator	PHAT Operator	HB Operator	Hannan–Thomson
Measured time difference	0.01520	0.01610	0.01515	0.01530	0.01515	0.01520
Absolute error	0.00020	0.00110	0.0005	0.0030	0.00015	0.00020

**Table 4:** Time-difference values determined in the test.

Time Difference	*t*_14_ (s)	*t*_24_ (s)	*t*_34_ (s)	*t*_54_ (s)	*t*_64_ (s)	*t*_74_ (s)	*t*_84_ (s)	Time (s)
Node backhaul time difference	0.02000	−0.00600	0.02850	−0.02800	−0.05575	0.00525	−0.02525	0.005
Software solution time difference	0.02019	−0.00602	0.02865	−0.02821	−0.05585	0.00503	−0.02533	750

**Table 5:** Results of location test.

Coordinate	x (m)	y (m)	z (m)	Error (m)
Actual location	43.21	−6.17	−9.12	—
Node backhaul time-difference positioning result	42.0625	−5.7140	−7.0896	1.3793
Software solution time-difference positioning result	42.2611	−6.2305	−8.6081	1.3850

## References

[1] Zhu Y.P., Deng B.W., Jiang A.M., Liu X.F., Tang Y.B., Yao X. (2018). ADMM-based TDOA Estimation. IEEE Commun. Lett..

[2] Wang Y., Ho K.C. (2017). TDOA Positioning Irrespective of Source Range. IEEE Trans. Signal Process..

[3] Salari S., Chan F., Chan Y.T., Read W. (2018). TDOA Estimation with Compressive Sensing Measurements and Hadamard Matrix. IEEE Trans. Aerosp. Electron. Syst..

[4] Xu Q.M., Li X., Chan C.Y. (2017). A Cost-Effective Vehicle Localization Solution Using an Interacting Multiple Model Unscented Kalman Filters (IMM-UKF) Algorithm and Grey Neural Network. Sensors.

[5] Lim J., Shin M., Hwang W. (2017). Variants of extended Kalman filtering approaches for Bayesian tracking. Int. J. Robust Nonlinear Control.

[6] Risoluti R., Gullifa G., Fabiano M.A., Wo L.W., Materazzi S. (2017). Biomimetic complexes of Cd(II), Mn(II), and Zn(II) with 2-aminomethylbenzimidazole. EGA/MS characterization of the thermally induced decomposition. Russ. J. Gener. Chem..

[7] Gan W.Y., Zhu D.Q., Ji D.X. (2018). QPSO-model predictive control-based approach to dynamic trajectory tracking control for unmanned underwater vehicles. Ocean Eng..

[8] Wen T.L., Jia Y., Huang D.Y., Lu K., Deng C., Zeng T., Yu S., He Z. (2018). Feature Extraction of Electronic Nose Signals Using QPSO-Based Multiple KFDA Signal Process. Sensors.

[9] Chave A.D. (2017). Estimation of the Magnetotelluric Response Function: The Path from Robust Estimation to a Stable Maximum Likelihood Estimator. Surv. Geophys..

[10] Lee K., Wu Y., Bresler Y. (2018). Near-Optimal Compressed Sensing of a Class of Sparse Low-Rank Matrices Via Sparse Power Factorization. IEEE Trans. Inf. Theory.

[11] Li Y.Y., Osher S. (2009). Coordinate descent optimization for l (1) minimization with application to compressed sensing; a greedy algorithm. Inverse Probl. Imaging.

[12] Wood S.U.N., Rouat J., Dupont S., Pironkov G., Wood S.U., Rouat J., Dupont S., Pironkov G. (2017). Blind Speech Separation and Enhancement With GCC-NMF. IEEE/ACM Trans. Audio Speech Lang. Process..

[13] Breslin C., Mani R.S., Fanta M., Hoch N., Weinfeld M., Caldecott K.W. (2017). The Rev1 interacting region (RIR) motif in the scaffold protein XRCC1 mediates a low-affinity interaction with polynucleotide kinase/phosphatase (PNKP) during DNA single-strand break repair. J. Biol. Chem..

[14] Gómez-Benito J., Berrío Á.I., Guilera G., Rojo E., Purdon S., Pino O. (2018). The Screen for Cognitive Impairment in Psychiatry: Proposal for a polytomous scoring system. Int. J. Methods Psychiatr. Res..

[15] Dam H.Q., Nordholm S. (2017). Source separation employing beamforming and SRP-PHAT localization in three-speaker room environments. Vietnam J. Comput. Sci..

[16] Zhang W.C., Wang S.F., Zhang Y.Z. (2018). Multiple-Model Adaptive Estimation with a New Weighting Algorithm. Complexity.

[17] Cordero E., Nicola F., Rodino L. (2010). Time-Frequency Analysis of Fourier Integral Operators. Commun. Pure Appl. Anal..

[18] Kumar S., Vig R., Kapur P. (2018). Development of Earthquake Event Detection Technique Based on STA/LTA Algorithm for Seismic Alert System. J. Geol. Soc. India.

[19] Singh V.P. (2017). Kinematic Wave Theory of Overland Flow. Water Resour. Manag..

[20] Jia Y.X., Chen G.B., Zhao P.F. (2017). Application of rigorous coupled-wave theory on quality analysis of natural jadeite. Glob. Geol..

[21] Oreshina M.N. (2017). Spectral decomposition of normal operator in real Hilbert space. Ufa Math. J..

[22] Wang C.Y., Gao Q., Wei R.J., Li T., Wang J.J. (2017). Spectral decomposition-based fast pressure integration algorithm. Exp. Fluids.

[23] Pan F., Song Q., Lin G.Y., Li L.L. (2016). Theoretical analysis and simulation of phase difference correction algorithm. Power Renew. Energy.

[24] Tu Y.Q., Shen Y.L. (2017). Phase correction autocorrelation-based frequency estimation method for sinusoidal signal. Signal Process.

[25] Zhang H., Lizana A., Iemmi C., Monroy-Ramírez F.A., Márquez A., Moreno I., Campos J. (2018). LCoS display phase self-calibration method based on diffractive lens schemes. Opt. Lasers Eng..

[26] Zhu X., Zhao H., Dong L., Wang H., Liu B., Yuan D., Tian A., Wang F., Zhang C., Ban X. (2017). High-precision self-adaptive phase-calibration method for wavelength-tuning interferometry. Meas. Sci. Technol..

[27] Biswas B., Mukherjee R., Chakrabarti I., Dutta P.K., Ray A.K. (2018). A High-Speed VLSI Architecture for Motion Estimation Using Modified Adaptive Rood Pattern Search Algorithm. Circuits Syst. Signal Process..

[28] Das S., Maity R., Maity N.P. (2018). VLSI-Based Pipeline Architecture for Reversible Image Watermarking by Difference Expansion with High-Level Synthesis Approach. Circuits Syst. Signal Process..

[29] Pourkhaatoun M., Zekavat S.A. (2011). High-resolution independent component analysis based time-of-arrival estimation for line-of-sight multipath environments. IET Commun..

[30] Quan Y.H., Li Y.C., Gao X.X., Meng D.X. (2016). FPGA Implementation of Real-Time Compressive Sensing with Partial Fourier Dictionary. Int. J. Antennas Propag..

[31] Vandenbussche J.J., Lee P., Peuteman J. (2014). Round-Off Noise of Multiplicative FIR Filters Implemented on an FPGA Platform. Appl. Sci..

